# Dr. Freer

**Published:** 1827-05

**Authors:** 


### OBITUARY.

Recently deceased, Dr. FREER, Professor of Medicine in Glasgow.

Died, on the 20th March, at his father's house in Torrington-square, in the twenty-fourth year of his age, of a pulmonary consumption, RALPH HENRY DUNKIN, member of the Royal College of Surgeons, only son of James William Dunkin, Esq.

He had applied with much earnestness to the study of anatomy and surgery, during a long term of pupillage to Mr. Travers, at St. Thomas's Hospital. In the course of a residence of some months at Paris, in 1826, for the purpose of further professional accomplishment, he appears to have contracted a complicated dyspepsia, which gradually laid the foundation for the fatal affection of the chest.

He was a young man of good understanding and quick attainment, of a truly benevolent heart, and amiable manners; and his early removal (premature, who shall say?) is regarded by the small circle of his professional friends as one of those frequent but serious losses to society, which it is spared the pain of knowing only because time was wanting to reveal it.

